# Effects of Mind-Body Qigong Exercise on Overall Health, Fatigue/Sleep, and Cognition in Older Chinese Immigrants in the US: An Intervention Study with Control

**DOI:** 10.1155/2024/2481518

**Published:** 2024-01-31

**Authors:** Jianghong Liu, Yi Yang, Clara Li, Adriana Perez, Adrian Raine, Haoer Shi, Liye Zou

**Affiliations:** ^1^University of Pennsylvania, Philadelphia, PA 19104, USA; ^2^Department of Psychiatry, Alzheimer's Disease Research Center, Icahn School of Medicine at Mount Sinai, New York, USA; ^3^Body-Brain-Mind Laboratory, School of Psychology, Shenzhen University, Shenzhen 518060, China

## Abstract

**Background:**

Culturally relevant exercises may help improve health and address disparities faced by older immigrants due to language and cultural barriers. Few studies have focused on such exercise interventions among older Chinese immigrants at US daycare centers.

**Methods:**

We conducted a 10-week nonrandomized controlled trial in older Chinese immigrants in Philadelphia, US. The intervention group practiced Chinese Qigong (Baduanjin) 5 days a week guided by trained research assistants and video instructions. The control group maintained their usual daily activities. We collected self-report assessments on overall health, sleep, and fatigue and implemented two computerized cognitive tests measuring psychomotor vigilance task (PVT) and memory twice, preintervention and postintervention. Repeated measures general linear model (GLM) and paired samples *t*-tests were used for data analyses.

**Results:**

Eighty-eight older adults (Qigong, *n* = 53; control, *n* = 35) with an average age of 78.13 (SD = 5.05) were included. Groups showed no significant differences at baseline evaluation. After the 10-week exercise, the intervention group showed significant improvements in overall health (*p*=0.032), fatigue (*p* < 0.001), and cognitive functions including memory (*p*=0.01), response speed (*p*=0.002), and response time (*p*=0.012) on the PVT, as well as marginally significant benefits in sleep (*p*=0.058). Between-group comparisons identified significant group-by-time interactions in health (*p*=0.024), sleep (*p*=0.004), fatigue (*p*=0.004), and memory (*p*=0.004).

**Conclusion:**

We revealed significant positive effects of Qigong in older Chinese immigrants across multiple health domains. Findings highlight the potential of a culturally relevant exercise in addressing health disparities.

## 1. Introduction

The aging population is confronted with a myriad of health conditions, such as cardiovascular diseases and diabetes[1, 2], which can lead to reduced quality of life. Sleep disturbances and fatigue are also common among the elderly, with studies reporting increased difficulty in initiating sleep [3] and daytime sleepiness [4]. Additionally, cognitive decline can have negative effects on daily functioning and independence [5]. Specifically, Asian Americans face numerous health challenges including cancer and cardiovascular conditions [6], as well as lower physical activity levels than other ethnic groups [7, 8], highlighting the importance of targeted healthcare interventions.

Numerous observational and intervention studies have documented the effectiveness of regular exercises in enhancing overall health, such as improving sleep [9], reducing chronic illness [10], and benefitting mental health [11]. Specific to the aging population, exercise has been demonstrated to improve their physical, mental, and cognitive health outcomes [12], including delaying cognitive decline [13]. Since culturally relevant exercises, such as Qigong, have been well-received by older Asian adults [14, 15], culturally relevant aspects could be incorporated into exercise programs to encourage physical activities and improve their health.

Mind-body exercises have demonstrated health benefits among older adults [16, 17]. Among such exercises, Qigong embodies the traditional Chinese philosophies by regulating yin and yang and *qi* flow throughout the body [18] and is thus less physically demanding for the elders to practice. Participants also benefit from the combination of physical activity, mindfulness, and breathing skills, which all contribute to overall health [19, 20]. Additionally, it is convenient to learn and practice, requiring minimal equipment and space. Qigong has been shown to reduce age-related cognitive decline among older adults [14, 21], improve their self-reported health status and quality of life [22], and reduce their stress and depression [22]. Baduanjin is a type of Qigong that works on the mind-body integration to regulate stress and emotion and enhance health. This exercise modality consists of 8 movements to stretch muscles and regulate blood flow to improve body functions. Baduanjin interventions have been shown to be feasible among older adults with low exercise-intensity tolerance due to the easy and slow movements [15] and have been documented to improve health outcomes [20, 23–25].

Baduanjin has increasingly been recognized as an effective antiaging approach, but it is not well-popularized and practiced worldwide. The majority of previous studies on Baduanjin intervention were conducted in China and mostly included Chinese participants living in their home country [26]. Very few studies have explored its beneficial effects among other Chinese populations, such as Chinese Americans or Chinese immigrants, whose lifestyles may be different from those residing in China [27, 28]. Moreover, the majority of the existing literature only focused on a single (or related) health domain, but few tested multiple health outcomes of an intervention program in the same study. Furthermore, studies have evaluated the role of daycare centers in supporting older adults' quality of life [29], but lack a central focus on exercise programs, particularly culturally relevant exercise interventions.

To address the gaps in the existing literature, we designed a nonrandomized controlled pilot trial aiming to test the multiple health effects of Baduanjin exercise among older Chinese adults in the US. We aimed to investigate whether the intervention group would show an improvement in physical and cognitive measures compared to the control group. Furthermore, since many factors affect older adults' health outcomes, such as age and gender [30], educational level [31, 32], marital status [33], number of children [34, 35], and BMI [36, 37], we also planned to control these potential confounding variables while testing the effects of the Qigong intervention.

## 2. Methods

### 2.1. Study Design

This was a two-group, nonrandomized controlled trial of an exercise (Qigong) intervention compared to routine daily activities. Assessments were taken at baseline and 10 weeks. We did not adopt a randomized controlled design since all participants attended the senior daycare center every day, thus making it very difficult to refrain participants in the control group from joining the daily exercise. Therefore, we started recruiting the control group several weeks after the intervention group completed all measurements.

### 2.2. Sample and Recruitment

We recruited participants who attended a senior daycare center in Chinatown, Philadelphia, in 2022. This facility hosted a total of 330 older Chinese American individuals, of whom 58% were female, and the majority were aged over 70 years. We included people who were greater than or equal to 65 years old, self-identified as Chinese, had a vision condition suitable for computerized cognitive testing, and were sufficiently physically healthy to participate in the exercise program. The exclusion criteria included having a very limited mobility level, severe psychiatric disorders, and other health conditions that hindered the performance of specific Baduanjin movements. The study was approved by the Institutional Review Board at the University of Pennsylvania (IRB #851589).

**Figure 1:**
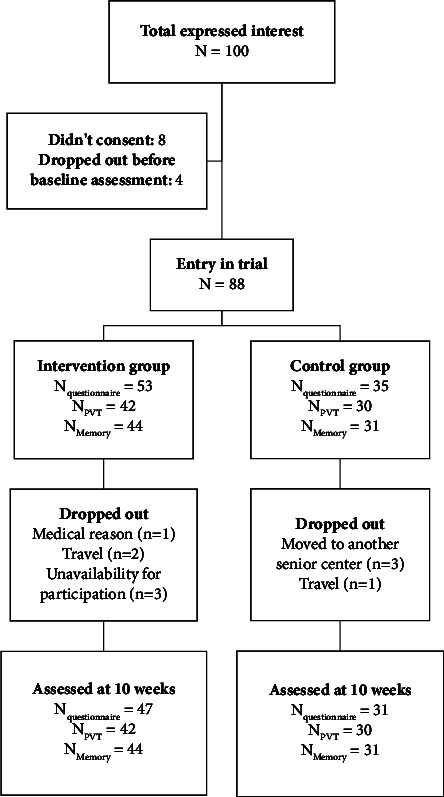
Consort flow diagram of participants in this study.

A total of 100 older adults expressed initial interest in the study, and 92 of them signed the written informed consent. Participants were assigned to the intervention and control groups based on their interests and availability. Specifically, we held the principle of “first come, first served,” so those who expressed interest first were assigned according to their preference for engaging in exercise or not. Additionally, participants having scheduling conflicts with the morning practice sessions were allocated to the control group. Despite the potential introduction of group bias due to self-selection, sociodemographic differences between the two groups were examined to ensure the absence of significant disparities. Of note, 4 participants dropped out before the first assessment due to the following reasons: (a) schedule conflict (*n* = 3) and (b) unwillingness to complete baseline questionnaires or cognitive tests (*n* = 1). As a result, 53 participants in the intervention group and 35 in the control group remained. During the intervention, 10 participants dropped out of the study (experimental group *N* = 6 and control group *N* = 4).  Figure 1 illustrates the CONSORT flowchart.

### 2.3. Procedure

The study procedures included obtaining consent, conducting preintervention (baseline) tests, intervention, and postintervention tests. The pretests and posttests each lasted two weeks. Participants who completed the self-report assessments and computerized cognitive tests received financial compensation. Before the formal 10-week intervention started, participants practiced the Baduanjin intervention protocol under the guidance of trained research assistants (RAs) by using a standard 12-minute video tutorial [38] of Baduanjin by the General Administration of Sport of China. A description of procedures was detailed in our previous baseline publication [15].

### 2.4. Intervention

The intervention group completed one set of Baduanjin Qigong exercises per day, five times per week (Monday to Friday) for 10 weeks in the auditorium of the senior daycare center following the video tutorial [38], with trained RAs demonstrating in front of participants. All participants in the intervention group practiced simultaneously. The Baduanjin exercise intervention protocol included a warm-up, an attendance collection, the 12-minute intervention, a postpractice movement correction, and a question-and-answer (Q&A) debrief, adding up to 30 minutes in total. Research team members arrived between 8:30 and 8:45 am each day to record attendance and attrition. The formal exercise session started at 9 am. When each exercise session finished, RAs corrected participants' exercise movements and answered their questions during the Q&A session. Additionally, RAs were responsible for documenting adverse events to monitor study-related injuries. Participants in the control group were advised to undertake routine activities.

### 2.5. Fidelity

In order to improve the reliability and validity of the intervention, the RAs received 6 hours of data collection training from the research team. We also manualized the study procedures to ensure consistency. Every exercise session adhered to the standard video tutorial to ensure consistent delivery of the intervention. RAs conducted presession checks on the technology devices and supervised participants' exercise movements. The principal investigator was present during 40% of sessions to assess the team's performance.

### 2.6. Measurements

Outcomes of interest in this study included overall health, physical health, and cognitive health. Trained RAs conducted the self-reported questionnaires in the library room at the senior daycare center and administered the cognitive tests in the computer room. The demographic questionnaire was designed by the research team, which included age, gender, educational background, occupation before retirement, marital status, number of children, medical history, and exercise frequency.

#### 2.6.1. Overall Health

The Subhealth Measurement Scale (SHMS V1.0) [39] was used to evaluate physical, mental, and social suboptimal health status with 39 items, each having 5 response categories for symptom frequency (1 = none and 5 = always). A higher total score indicates better health status. The SHMS V1.0 had a Cronbach's *α* of 0.921 [40] and attained a Cronbach's *α* of 0.901 and 0.913 in the current study, at pretest and posttest, respectively.

#### 2.6.2. Physical Health

*(1) Sleep Quality*. Sleep quality was assessed using the Pittsburgh Sleep Quality Index (PSQI) [41]. It contains 18 questions covering seven components, including subjective sleep quality, sleep latency, and sleep duration. Greater total scores indicate poor sleep quality. Cronbach's *α* of the Chinese version of the PSQI was previously reported to be 0.82–0.83 [42], which is consistent with the current study showing a Cronbach's *α* of 0.818 at the pretest and 0.806 at the posttest.

*(2) Fatigue*. Fatigue was assessed with the Fatigue Severity Scale (FSS) [43], which contains 9 items that evaluate fatigue severity over a period of one week. Each item is scored from 1 (completely disagree) to 7 (completely agree), and a higher score indicates a more severe fatigue level. The Chinese version of FSS had a Cronbach's *α* of 0.95 [44]. Cronbach's *α* in the current study was 0.917 at the pretest and 0.946 at the posttest.

#### 2.6.3. Cognitive Health: Computerized Cognitive Tests

*(1) Memory*. A total of 90 pictures, consisting of landscapes, objects, and nonfamous people randomly selected from the International Affective Pictures System (IAPS) [45], were presented on the computer screen. Participants were asked to remember as many pictures as possible. A few minutes after encoding, a new set of pictures was given to participants for them to decide whether it was from the first set (press F button) or not (press J button). We measured participants' performance using hit rate, correct rejection, misses, false alarms, and the D-prime (d') score (*z*-transformed hit rates minus *z*-transformed false alarm rates). These measurements help assess participants' performance on visual memory.

*(2) Alertness and Vigilance*. The psychomotor vigilance test (PVT) was used to evaluate attention and response to visual stimuli [46]. During the 10-minute test, participants were instructed to press a button when a number appeared on the computer screen. We recorded participants' response time and speed as measurements of alertness and vigilance. A shorter response time indicates higher vigilance.

### 2.7. Statistical Analysis

All analyses were performed using SPSS, Version 26 (IBM, Chicago, IL). Means, standard deviations (SDs), and ranges were used to describe the participants' demographic characteristics. The normality of the data and potential outliers were examined. Independent samples *t*-tests and chi-square tests were used to evaluate the parity between intervention and control groups on baseline demographic variables. For each continuous outcome variable, a 2 (group: intervention versus control) by 2 (time: pretest versus posttest) repeated measures analysis of covariance (ANCOVA) was conducted to investigate the intervention effects. For categorical or ordinal outcomes, a generalized estimating equations (GEE) model with group and time as fixed effects and subjects as random effects were performed. All models were controlled for age, gender, education, occupation before retirement, marital status, children status, and BMI, and sleep-relevant models were additionally controlled for nap frequency. The BMI was calculated based on weight and height information obtained from the nurses at the daycare center who periodically tracked the elders' health condition. Interactions were broken down using paired samples *t*-test or Wilcoxon signed-rank tests, for continuous or categorical variables, respectively, to evaluate the potential within-group pretest/posttest differences. A *p* value of <0.05 was considered significant, while a *p* value larger than 0.05 but smaller than 0.10 was regarded as marginally significant. Two-tailed tests were employed throughout.

## 3. Results

### 3.1. Demographics

Overall, 88 participants (female: 85.2%) aged between 66 and 93 years (mean = 78.13 and SD = 5.05) were included for the data analysis. Four participants from the control group were removed as outliers in the analysis of the memory d' score due to careless responses. The mean ages of participants in the intervention group (*n* = 53) and control group (*n* = 35) were 78.43 (SD = 4.98) and 77.66 (SD = 5.18) years, respectively. For both groups, the majority (intervention: 88.7% and control: 80%) of the participants were female. Significant group differences in demographic variables were not observed (Table 1). We also collected data on the activity level for both groups, and the control group did not show a significant difference between the pretest and posttest.

### 3.2. Health Outcomes

#### 3.2.1. Subhealth Measurement Scale (SHMS)

**Figure 2:**
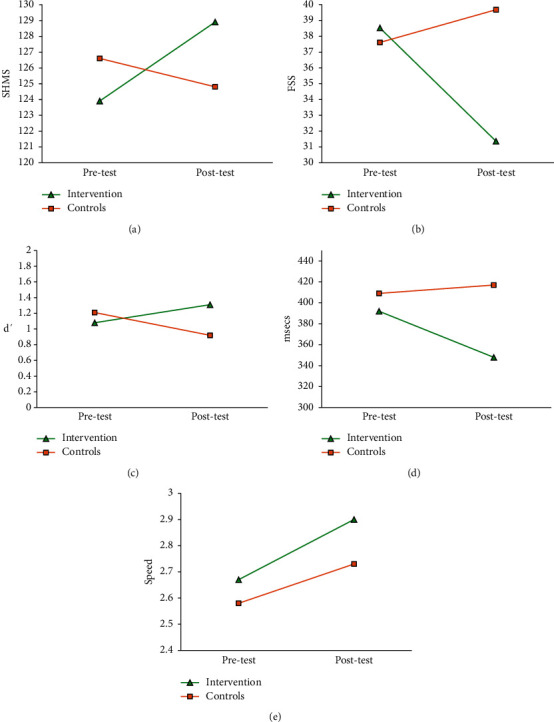
Baduanjin intervention effects: (a) overall health (SHMS), (b) self-report fatigue (FSS), (c) memory (d'), (d) psychomotor vigilance (response time), and (e) psychomotor vigilance (response speed).

Overall health was found to be significantly improved after the intervention (group × time interaction: *F* (1, 64) = 5.336, *p*=0.024, Figure 2(e)). The intervention group exhibited a significant increase in the SHMS health score (*t* = −2.21, *p*=0.032, *d* = −0.33). In the control group, a nonsignificant decreasing trend was found (*t* = 0.75, *p*=0.46, *d* = 0.14) (for details, see Figure 2).

#### 3.2.2. Sleep (PSQI)

Overall, the intervention improved sleep in terms of the proportion of good (PSQI ≤5) and poor (PSQI >5) sleepers, with a significant group × time interaction effect being observed (*χ*^2^=8.514 and *p*=0.004). The intervention group showed marginally significant improvement from 29.8% to 42.6% (*Z* = −1.897 and *p*=0.058). In contrast, the control group experienced a significant decrease in the percentage of good sleepers from 25.8% to 12.9% (*Z* = −2.000 and *p*=0.046). A marginal significant interaction effect (*F* (1, 66) = 3.147 and *p*=0.081) was also observed for the continuous PSQI score, despite no significant within-group differences being identified (for details, see Figure 2).

#### 3.2.3. Fatigue (FSS)

The fatigue was substantially improved after the Baduanjin intervention, with a significant group × time interaction effect (*F* (1, 66) = 8.771 and *p*=0.004; Figure 2(d)). Decomposition of the interaction demonstrated a significant decrease in fatigue in the intervention group (*t* = 3.55 and *p* < 0.001, *d* = 0.52). Conversely, a nonsignificant increasing trend (*t* = −0.87, *p*=0.389, and *d* = −0.16) was observed in the control group. For details, see Figure 2.

### 3.3. Cognition Outcomes

Detailed results of within-group and between-group comparisons are summarized in Tables 2 and 3, respectively.

#### 3.3.1. Memory (d')

Memory performance was significantly improved after intervention, with the repeated measures GLM analysis identifying a significant group × time interaction (*F* (1, 59) = 8.771 and *p*=0.004; Figure 2(a)) on memory d' score. The paired samples *t*-test revealed a significant increase in d' score (*t* = −2.53, *p*=0.015, and *d* = 0.38) in the intervention group, whereas the control group showed a significant decline in d' score (*t* = 2.76, *p*=0.010, and *d* = −0.38).

#### 3.3.2. Psychomotor Vigilance

*(1) Response Speed*. The intervention group demonstrated a significant increase in response speed (*t* = −3.24, *p*=0.002, and *d* = 0.41; Figure 2(b)). The control group similarly showed a marginally significant improvement (*t* = −1.98, *p*=0.057, and *d* = 0.50), possibly indicating the learning effects of the vigilance task at repeated exposure. For the between-group comparison, no significant group, time, or group × time effect was observed.

*(2) Reaction Time*. The intervention group's reaction time significantly decreased after training (*t* = 2.63, *p*=0.012, and *d* = 0.41; Figure 2(c)), while the control group showed no significant changes over time (*t* = 0.51, *p*=0.611, and *d* = 0.41). However, the main effects of group, time, and the group × time interaction in the between-group analysis were nonsignificant.

## 4. Discussion

New immigrants often face health disparities due to obstacles within the United States' health services [47]. Immigrant status, language barriers, and cultural differences all create barriers to appropriate and timely care [48]. Additionally, many immigrants are reluctant to join mainstream physical activities (for example, endurance, recreation, and sports activities) and exhibit a low participation in exercises [49]. There lacks a central focus on exercise programs, particularly culturally relevant exercise interventions among new immigrants, at senior daycare centers in the US. In this intervention study, we implemented the Baduanjin Qigong exercise intervention among older Chinese immigrants in the US. The key finding of our study was that the 10-week Baduanjin exercise intervention could improve older adults' physical and cognitive health.

The improvements observed in our intervention program were in general consistent with previous studies [26]. For example, multiple systematic reviews of randomized controlled trials have identified Baduanjin Qigong's benefits to older adults, including improving global cognitive function and specific domains of cognition [21, 24, 50] and enhancing muscle strength, balance, and cardiorespiratory fitness [23], as well as reducing falls and pain [51]. Nevertheless, there were some mixed results in the prior literature as well, with some researchers not finding statistically significant positive outcomes of mindfulness-based training [52]. This could be due to the lack of fidelity and compliance of training, or the lack of culturally tailored components of exercise in respect to the study population. In contrast, our culturally relevant exercise intervention had relatively good attendance and low attrition rates, which may partly explain the significant positive results observed.

Interestingly, both the length of our exercise program and the duration of each session were relatively short compared to previous studies. Many of the previous intervention trials on physical health outcomes have lasted more than 12 weeks, and those on cognitive health usually lasted even longer, around 6 months, with each session ranging from 20 to 45 minutes [20]. On the other hand, our pilot intervention lasted for 10 weeks, 30 minutes per day only, but has led to significant improvements in both physical and cognitive health domains among the older adults. This suggests that even a relatively short period of exercise intervention can result in positive health changes, as seen in previous mindfulness-based intervention studies as well [53, 54]. The reduced demands from a shorter duration and length of the intervention may enhance compliance and motivation compared to longer programs. Future research is needed to see if the health benefits still remain after longitudinal follow-ups.

Although the specific biological mechanisms under which exercise interventions improve health are not fully understood, several potential pathways were reported. For example, physical training could lead to changes in the cerebrovascular system, which is associated with cognitive performance improvements [55]. Additionally, physical activities may facilitate functional connectivity in the prefrontal cortex [56] and left parietal lobe [57] and increase the grey matter volume of the left medial frontal gyrus [58], all key regions related to cognitive health. Specific to Baduanjin, the combination of mindfulness, physical movements, and breathing techniques [59, 60] helps regulate *qi* (the vital energy in the human body) and blood flow (the supplier of *qi*) in the individual's body and further activates various critical acupoints [61]. Additionally, Baduanjin exercise may increase the grey matter volume in the temporal lobe [62] and lead to a better dorsal attention network [63], which is important in regulating cognitive functions. Finally, the fully in-person group exercise sessions may have served as a social connection for the participants, which could also be related to better cognitive functioning [64].

Our study has several strengths. First, most previous studies on Baduanjin Qigong included only Chinese individuals from China, but our study recruited Chinese immigrants from the US to demonstrate the effectiveness of this culturally relevant exercise program in this population. Second, we employed objective computerized measurements to assess the cognitive functions of older adults, which could be more accurate than subjective, self-report measures. Nevertheless, several limitations of our study should also be noted. First, we recruited only 88 Philadelphia-based older Chinese immigrants in the study, so the results of this small, localized sample might not fully generalize to wider populations. Second, the effect of our exercise intervention was only evaluated for the 10-week study phase without long-term follow-ups. Therefore, we could not conclude whether Baduanjin has lasting beneficial effects on older adults' health. Third, in order to make our intervention program culturally relevant, we only recruited participants who identified as ethnically Chinese. It is thus unknown if Baduanjin is effective in improving the health outcomes of other ethnic groups. Fourth, the duration of our tutorial was relatively short, and we did not collect biometric data. Future studies could extend the exercise duration and investigate how biometric measures, such as heart rate, were affected by the exercise intervention. Finally, we did not blind the data collectors in this pilot study, which may potentially induce biases. However, all questionnaires were self-reported by participants, and all cognitive tests were objective and computerized, thus they should not be subjected to the tester's biases.

## 5. Implications and Conclusions

Baduanjin Qigong exercise could be used in health promotion due to its ease of implementation and its short-time frame to yield positive health results. At the individual level, community-dwelling older adults could participate in Baduanjin daily exercise to enhance their physical and cognitive health conditions. At the community level, institutions and communities can collaboratively develop community-wide Baduanjin exercise programs as preventive strategies for physical health issues and age-related cognitive decline.

Longitudinal randomized controlled trials should be conducted to further test the efficacy of the Baduanjin exercise. Additionally, the in-person group exercise could be extended to home-based formats [65], which would make the exercise more accessible. Furthermore, individuals from different ethnic groups could be included in the exercise intervention to test if Baduanjin could improve health for a diverse population.

In conclusion, our study involved the intervention of the Baduanjin exercise at a senior daycare center in Philadelphia. We gathered the pretest and posttest data and implemented group exercises. Baduanjin was shown to be effective in improving physical and cognitive health in older adults. Our study may shed light on the development of culturally tailored interventions addressing physical and cognitive health issues, especially dementia and mild cognitive impairment (MCI) for new immigrants and underrepresented older adults. Future research with larger sample sizes from diverse populations and employing a more robust randomized controlled trial design is warranted to extrapolate and validate the current findings.

## Figures and Tables

**Table 1:** Demographics of intervention and control groups.

	Total (*N* = 88)	Intervention (*N* = 53)	Control (*N* = 35)	*t*/*χ*^2^	*P* value
Age, mean (*SD*) (range)	78.13 (5.05) (66 to 93)	78.43 (4.98) (69 to 93)	77.66 (5.18) (66 to 88)	0.705	0.483
*Gender, N (%)*				1.261	0.261
Male	13 (14.8)	6 (11.3)	7 (20.0)		
Female	75 (85.2)	47 (88.7)	28 (80.0)		
*Educational level, N (%)*				5.947	0.203
Primary school and below	14 (15.9)	10 (18.9)	4 (11.4)		
Middle school	19 (21.6)	11 (20.8)	8 (22.9)		
High school	15 (17.0)	11 (20.8)	4 (11.4)		
Vocational school	9 (10.2)	7 (13.2)	2 (5.7)		
University or college and above	31 (35.2)	14 (26.4)	17 (48.6)		
*Occupation before retirement, N (%)*				10.799	0.148
Civil servant	6 (6.8)	3 (5.7)	3 (8.6)		
Office worker	17 (19.3)	10 (18.9)	7 (20.0)		
Specialist	40 (45.5)	19 (35.8)	21 (60.0)		
Business personnel	3 (3.4)	3 (5.7)	0 (0.0)		
Private business owner	2 (2.3)	2 (3.8)	0 (0.0)		
Housewife	2 (2.3)	1 (1.9)	1 (2.9)		
Freelancer	3 (3.4)	3 (5.7)	0 (0.0)		
Other occupation	15 (17.0)	12 (22.6)	3 (8.6)		
*Marital status, N (%)*				5.158	0.271
Never married	2 (2.3)	0 (0.0)	2 (5.7)		
Married	56 (63.6)	32 (60.4)	24 (68.6)		
Divorced	2 (2.3)	1 (1.9)	1 (2.9)		
Widowed	27 (30.7)	19 (35.8)	8 (22.9)		
Separated	1 (1.1)	1 (1.9)	0 (0.0)		
BMI (kg/m^2^), mean (SD) (range)	24.81 (3.43) (16.77 to 35.49)	24.97 (3.20) (17.19 to 33.71)	24.55 (3.83) (16.77 to 35.49)	0.540	0.590
*Nap frequency, N (%)*				6.199	0.102
Never	18 (22.2)	8 (16.0)	10 (32.3)		
1-2 times/week	13 (16.0)	6 (12.0)	7 (22.6)		
3-4 times/week	14 (17.4)	11 (22.0)	3 (9.7)		
5–7 times/week	36 (44.4)	25 (50.0)	11 (35.5)		

**Table 2:** Within-group effects on cognition and self-report health outcomes.

Variable	Pretest mean (SD)	Posttest mean (SD)	Paired differences (95% CI)	*t*/*Z*	*P* value
*Overall health (SHMS) (N*_*i*_=45, *N*_*c*_=31)					
Intervention	123.96 (15.96)	128.89 (16.51)	−4.933 (−9.424 to −0.443)	−2.214	**0.032**
Control	126.58 (15.86)	124.81 (17.53)	1.774 (−3.059 to 6.608)	0.750	0.459
*Subjective sleep report (PSQI) (N*_*i*_=47, *N*_*c*_=31)					
Intervention	8.43 (4.74)	7.55 (4.67)	0.872 (−0.356 to 0.2.101)	1.429	0.160
GS, *N* (%)	14 (29.8)	20 (42.6)	—	−1.897	0.058
PS, *N* (%)	33 (70.2)	27 (57.4)			
Control	10.26 (5.32)	10.13 (4.81)	0.129 (−1.087 to 1.345)	0.217	0.830
GS, *N* (%)	8 (25.8)	4 (12.9)	—	−2.000	**0.046**
PS, *N* (%)	23 (74.2)	27 (87.1)			
*Self-report fatigue (FSS) (N*_*i*_=47, *N*_*c*_=31)					
Intervention	38.53 (11.22)	31.36 (11.05)	7.170 (3.107 to 11.233)	3.552	**0.001**
Control	37.61 (12.86)	39.68 (14.31)	−2.065 (−6.892 to 2.763)	−0.873	0.389
*Memory (d') (N*_*i*_=44, *N*_*c*_=27)					
Intervention	1.08 (0.729)	1.31 (0.721)	−0.229 (−0.413 to −0.046)	−2.527	**0.015**
Control	1.21 (0.615)	0.917 (0.655)	0.293 (0.075 to 0.512)	2.758	**0.010**
*Vigilance (response speed) (N*_*i*_=42, *N*_*c*_=30)					
Intervention	2.67 (0.592)	2.90 (0.441)	−0.221 (−0.359 to −0.0834)	−3.240	**0.002**
Control	2.58 (0.544)	2.73 (0.586)	−0.156 (−0.317 to −0.005)	−1.983	0.057
*Vigilance (response time) (N*_*i*_=42, *N*_*c*_=30)					
Intervention	391.67 (122.52)	348.31 (62.30)	43.36 (10.12 to 76.59)	2.635	**0.012**
Control	418.67 (203.25)	406.50 (265.28)	12.17 (−36.21 to 60.54)	0.514	0.611

SD, standard deviation; *N*_*i*_, number of samples in the intervention group; *N*_*c*_, number of samples in the control group; *Z*, Wilcoxon signed-rank test statistics; d', *z*-transformed hit rates minus *z*-transformed false alarm rates (higher rates indicate better memory); PSQI, higher scores indicate more sleep problems; SHMS, higher scores indicate better health; FSS, higher scores indicate more severe fatigue; GS, good sleeper (PSQI score ≤5); PS, poor sleeper (PSQI score >5). The bold values indicate the significant p values.

**Table 3:** Between-group comparison on cognition and self-report health outcomes.

	Group		Time		Group × time	
	*F*	*P* value	*F*	*P* value	*F*	*P* value
Health (SHMS)	1.753	0.190	0.285	0.595	5.336	**0.024**
Sleep (PSQI)	5.403	0.023	0.001	0.997	3.147	**0.081**
Good/poor sleeper^*∗*^	3.476	0.062	0.216	0.642	8.514	**0.004**
Fatigue (FSS)	3.055	0.085	0.374	0.543	7.290	**0.009**
Memory (d')	0.680	0.413	0.045	0.833	8.771	**0.004**
Vigilance (speed)	3.493	0.066	2.120	0.151	0.002	0.961
Vigilance (RT)	2.653	0.108	0.961	0.331	0.073	0.788

SD, standard deviation. Models are controlled for age, gender, education, occupation before retirement, marital status, and BMI, and sleep-relevant models are additionally controlled for nap frequency. ^*∗*^Results from the generalized estimating equations (GEE) model on a binary variable of a good or poor sleeper. Good sleeper, PSQI score ≤5. Poor sleeper, PSQI score >5. Type III Wald chi-square statistics were reported. The bold values indicate the significant p values.

## Data Availability

The data used to support the findings of this study are available from the corresponding author upon request.
